# Allele-specific PCR with fluorescently labeled probes: criteria for selecting primers for genotyping

**DOI:** 10.18699/vjgb-24-40

**Published:** 2024-06

**Authors:** V.A. Devyatkin, A.A. Shklyar, A.Zh. Fursova, Yu.V. Rumyantseva,, O.S. Kozhevnikova

**Affiliations:** Institute of Cytology and Genetics of the Siberian Branch of the Russian Academy of Sciences, Novosibirsk, Russia; Institute of Cytology and Genetics of the Siberian Branch of the Russian Academy of Sciences, Novosibirsk, Russia; Institute of Cytology and Genetics of the Siberian Branch of the Russian Academy of Sciences, Novosibirsk, Russia; Institute of Cytology and Genetics of the Siberian Branch of the Russian Academy of Sciences, Novosibirsk, Russia; Institute of Cytology and Genetics of the Siberian Branch of the Russian Academy of Sciences, Novosibirsk, Russia

**Keywords:** genotyping, single-nucleotide polymorphisms, TaqMan probes, LNA modifications, allele-specific PCR, генотипирование, однонуклеотидные полиморфизмы, зонды TaqMan, LNA-модификации, аллель-специфичная ПЦР

## Abstract

Single-nucleotide polymorphisms (SNPs) can serve as reliable markers in genetic engineering, selection, screening examinations, and other fields of science, medicine, and manufacturing. Whole-genome sequencing and genotyping by sequencing can detect SNPs with high specificity and identify novel variants. Nonetheless, in situations where the interest of researchers is individual specific loci, these methods become redundant, and their cost, the proportion of false positive and false negative results, and labor costs for sample preparation and analysis do not justify their use. Accordingly, accurate and rapid methods for genotyping individual alleles are still in demand, especially for verification of candidate polymorphisms in analyses of association with a given phenotype. One of these techniques is genotyping using TaqMan allele-specific probes (TaqMan dual labeled probes). The method consists of real-time PCR with a pair of primers and two oligonucleotide probes that are complementary to a sequence near a given locus in such a way that one probe is complementary to the wild-type allele, and the other to a mutant one. Advantages of this approach are its specificity, sensitivity, low cost, and quick results. It makes it possible to distinguish alleles in a genome with high accuracy without additional manipulations with DNA samples or PCR products; hence the popularity of this method in genetic association studies in molecular genetics and medicine. Due to advancements in technologies for the synthesis of oligonucleotides and improvements in techniques for designing primers and probes, we can expect expansion of the possibilities of this approach in terms of the diagnosis of hereditary diseases. In this article, we discuss in detail basic principles of the method, the processes that influence the result of genotyping, criteria for selecting optimal primers and probes, and the use of locked nucleic acid modifications in oligonucleotides as well as provide a protocol for the selection of primers and probes and for PCR by means of rs11121704 as an example. We hope that the presented protocol will allow research groups to independently design their own effective assays for testing for polymorphisms of interest.

## Abstract (ru)

Однонуклеотидные полиморфизмы (SNP) могут служить надежными маркерами в генной инженерии, селекции, скрининговых обследованиях и других областях науки, медицины и производства. Полногеномное секвенирование и генотипирование при помощи секвенирования могут высокоспецифично детектировать SNP и выявлять новые аллели. Однако в ситуациях, когда интерес исследователей направлен на отдельные конкретные локусы, эти методы становятся избыточными, а их цена, доля ложноположительных и ложноотрицательных результатов и трудозатраты на пробоподготовку и анализ не оправдывают их применения. Поэтому точные и быстрые методы генотипирования отдельных аллелей все еще остаются востребованными, особенно при проверке кандидатных полиморфизмов в анализах ассоциации с определенным фенотипом. Один из таких методов – генотипирование с использованием аллель-специфичных зондов TaqMan (TaqMan dual labeled probes). Метод заключается в реакции ПЦР в реальном времени с использованием пары праймеров и двух олигонуклеотидных зондов, комплементарных последовательности вблизи данного локуса таким образом, что один зонд комплементарен аллелю дикого типа, а другой – мутантному аллелю. Преимущества метода заключаются в его специфичности, чувствительности, невысокой стоимости и быстроте получения результатов. Он позволяет с высокой точностью различать аллели в геноме в одностадийной ПЦР без дополнительного этапа разделения продуктов реакции, что делает его востребованным в исследованиях генетических ассоциаций в молекулярной генетике и медицине. Благодаря развитию технологий синтеза олигонуклеотидов и совершенствованию методов подбора праймеров и зондов можно ожидать расширения возможностей применения этого подхода в диагностике наследственных заболеваний. В настоящей статье мы разобрали основные принципы метода, процессы, влияющие на результат генотипирования, критерии подбора оптимальных праймеров и зондов, использование LNA-модификаций в олигонуклеотидах, а также привели протокол подбора праймеров, зондов и ПЦР на примере SNP rs11121704. Мы надеемся, что представленный протокол позволит исследовательским группам самостоятельно подбирать собственные эффективные тест-системы для проверки интересующих полиморфизмов

## Introduction

Single-nucleotide polymorphisms (SNPs) are actively used as reliable markers in genetic engineering, selection, screening examinations, and other fields of science, medicine, and industrial production. It is clear that whole-genome sequencing and genotyping by sequencing can detect SNPs with high specificity and identify novel variants. On the other hand, in situations where the interest of researchers is focused on individual specific loci, these methods become redundant, and their cost, the proportion of false positive and false negative results, and the labor costs for sample preparation and analysis do not justify their use. Accordingly, accurate and rapid techniques for genotyping individual alleles are still in demand, especially for verification of candidate polymorphisms in analyses of association with a given phenotype (Kalendar et al., 2022).

Currently, methods based on allele-specific PCR allow to obtain the most accurate results at a low cost and do not require highly qualified personnel or expensive laboratory equipment. In this work, we analyze principles of work with one of these approaches: genotyping by means of allele-specific probes based on the TaqMan method (TaqMan dual labeled probes). It was first described 15 years ago (Hui et al., 2008) and remains one of the most popular for the detection of SNPs. The accuracy of the method ensures genotyping error of less than one case per 2,000 (Ranade et al., 2001). The correct choice of primers and probes is possible for most genome sequences and in more than 90 % of cases allows to obtain fairly accurate genotyping results for high-quality DNA without further optimization.

The method of allele-specific PCR with TaqMan probes can separate genotypes even with small amounts of an initial sample, does not require post-PCR processing, and correlates well with other methods (Broccanello et al., 2018). Nonetheless, kits that are commercially available and developed for a specific SNP are expensive, and recommendations for creating and optimizing one’s own assays are described rather superficially in most of literary sources.

In this work, we provide a detailed analysis of this technique with a description of processes that can influence genotyping results as well as recommendations for designing your own assays.

## Description of the method

The method consists of real-time PCR involving a pair of primers (forward and reverse, between which a polymorphic locus of interest is located) and two oligonucleotide probes complementary to a sequence near this locus in such a way that one probe is complementary to the wild-type allele, and the other to a mutant one. Each probe has a distinct fluorescent dye at the 5′ end and a fluorescence quencher and phosphate group at the 3′ end. The phosphate group prevents the probes from acting as primers in the PCR. Due to the proximity of the quencher and dye, an intact probe does not yield a signal because of Förster resonance energy transfer (FRET) and fluorescence quenching. At the elongation stage, a Taq DNA polymerase molecule that has reached the probe bound to the fully complementary template hydrolyzes it owing to 5′–3′ exonuclease activity, thereby uncoupling the quencher and dye, and the fluorescence is detected by an instrument.

Hybridization of the probe with the template is more effective in the case of complete complementarity; moreover, in the case of an unpaired base (mismatch), when a probe corresponding to one allele binds to the template corresponding to another allele, the polymerase preferentially displaces it entirely without separating the chromophores. Therefore, signal accumulation will occur much more efficiently in the case of complete complementarity between the probe and the template. Thus, the ratio of fluorescence levels of the different dyes depends on the ratio of the alleles (corresponding to the probes labeled with these dyes) in the initial template (Hui et al., 2008).

**Fig. 1.**
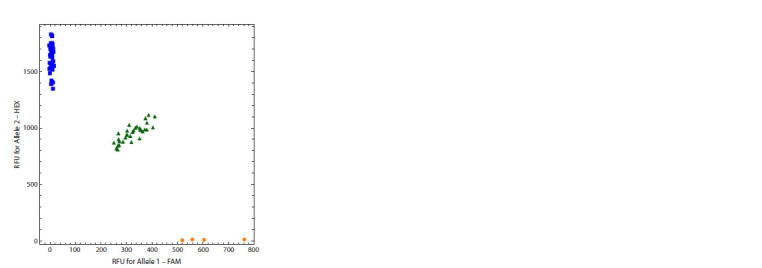
A high-quality allelic discrimination plot Here and in Fig. 2: Created in Bio-Rad CFX Manager software. RFU: relative fluorescence units.

In a situation close to ideal, an allelic discrimination plot, where X- and Y-axes correspond to the fluorescence levels of the first and second dye for each sample, looks like the one in Figure 1. Samples having the same genotype form a cloud of dots distant from other clusters. The fluorescence level of each dye is zero for homozygotes that do not have the allele labeled with this dye and is almost twice as high for homozygotes of a given allele as compared to heterozygotes

**Fig. 2:**
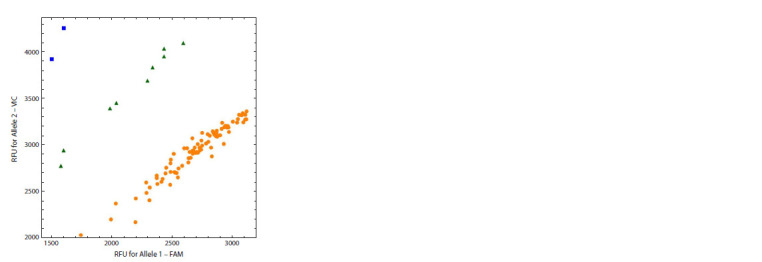
The allelic discrimination plot with a low ratio of target fluorescence to the background signal, but reliable discrimination of the genotypes is still possible.

In Figure 2, an outcome of a less specific reaction is shown, when annealing and subsequent restriction of probes additionally take place on the template corresponding to the other allele. Under this scenario, fluorescence intensity of both dyes is non-zero for all samples. On the other hand, the genotypes of the samples can still be well discriminated with high accuracy. It should be noted that during the reaction, the concentration of the specific probe decreases, the concentration of the template increases, and the probe with the mismatch remains intact, which shifts the equilibrium toward the formation of a duplex containing the mismatch. In this case, using a smaller amount of the initial template in the reaction or reading an allelogram at earlier cycles can help.

Based on the ratio between the dye signals in a single sample, it is impossible to directly determine which probe binds better and, accordingly, what the genotype is, because the dyes have different fluorescence intensities, probes differ in binding efficiency, and this process can be influenced by other random factors. Therefore, for the assay, it is necessary to employ several samples, among which there are different genotypes. It is recommended to analyze at least 20 samples per instrument run for reliable discrimination. By means of how much one signal increases more intensely than the other (i. e., by means of the angle between the X- and Y-axes in the plot for each sample), you can separate all samples into groups, and if three groups are detected, you can be confident in accurate determination of the genotype of each group. When assessing the results in the absence of a certain genotype among the samples, one should rely on the match between the actual frequency of genotypes and the one expected according to the literature or the Hardy–Weinberg equilibrium. To ensure correct allelic discrimination, it is advisable to verify samples of each genotype by Sanger sequencing.

## Criteria for designing optimal primers and probes for genotyping

Design of primers

– GC content within 30–80 % (ideally 40–60 %). – Stretches of a repeated nucleotide, especially four or more consecutive Gs, should be avoided. – Melting point (Tm ) in the range of 58–60 °C. The difference in Tm between the forward and reverse primers is no more than 2 °C. – Among the five nucleotides at the 3′ end, more than two Gs and/or Cs are not recommended. T should be avoided at the 3′ end. G or C at the last position at the 3′ end is a more suitable binding site for DNA polymerases. – Length 18–30 nucleotides. – The primers and probes must not overlap. – The recommended amplicon length according to the literature is 80–120 nucleotides.

Increasing the PCR product length reduces reaction efficiency and nuclease activity of Taq polymerase (Debode et al., 2017). In some cases, depending on nucleotide composition, to meet other criteria, amplicon length can be increased to 1,000. The minimum length is determined by the total length of the primers and probes. It is best to keep the distance between a probe and the primer annealing to the same strand shorter to the extent feasible, no more than 20 nucleotides if possible. Our experience shows that amplicon length of 98 to 469 bp and a 22–348 bp distance from a probe to the primer complementary to the same strand have no visible effect on discrimination accuracy.

From the point of view of simplicity and low cost of the experiment, we recommend selecting primers by taking into account the possibility of Sanger sequencing from the same primers. In this case, it is desirable that there be at least 50 nucleotides from the sequencing primer to the polymorphic site and from the polymorphic site to the other end of the maximal read (i. e., the regions upstream and downstream of the polymorphic site).

Design of probes

– Both probes must anneal to the same strand and not be complementary to each other. – Tm should be approximately 6–8 °C higher than that of the primers (as opposed to qPCR involving only one probe, for which the Tm should be 10 °C higher than that of the primers). The reason is that as the reaction mixture cools, the oligonucleotide probes must anneal to the DNA template before the primers do. – The greater the difference in Tm between the probe fully paired with the template and the probe carrying an unpaired base, the more efficiently can the alleles be separated. The minimum difference that has allowed us to separate alleles is 3 °C, but we advise designing probes to achieve a minimum of 4–5 °C whenever possible. The wider the temperature window, the easier it is to select an annealing temperature at which the annealing probability of a probe carrying a mismatch is negligible compared to that of a fully complementary probe. – Do not place G at the 5′ end because it will quench the fluorophore attached to it after cleavage the probe. Furthermore, Taq polymerase cleaves such probes worse (Huang, Li, 2009). The cleavage of the oligonucleotide begins with the appearance of 1–2 unpaired nucleotides at the 5′ end, which are recognized by the nuclease domain. An unpaired G at the end dramatically disrupts complementarity, thereby sometimes causing complete strand separation faster than Taq polymerase can begin to cut the probe; thus, the probe is displaced entirely as if it was not fully complementary to begin with. – Of the two strands, choose one such that the probes contain more Cs than Gs because empirical data indicate that such probes are more likely to produce a strong signal (https:// www.thermofisher.com/order/catalog/product/450025). – The polymorphic site should be located approximately in the middle third of the probe. – GC content within 20–80 % (ideally 30–70 %). – It is advisable to select the positions of the start and end of each probe so that Tm for both probes becomes approximately the same. – The length of the probes is 18–30 nucleotides, and the optimal length is 20 nucleotides. These restrictions are due to the fact that a probe must bind specifically to only one region within the amplified fragment and satisfy Tm requirements. The longer the entire probe, the smaller is the contribution of the polymorphic site to the melting temperature, the smaller is the percentage difference in Tm for cases of complete complementarity and mismatch, and less effectively are the alleles discriminated. A length greater than 30 nucleotides is acceptable, but in such cases, the quencher should not be located at the 3′ end but inside the probe at a distance of approximately 18–25 nucleotides from the 5′ end. The reason is that at distances between the dye and quencher greater than 100 Å (corresponding to approximately 30 bp in B-form DNA structure), FRET is disrupted and intact probes can emit fluorescence, thus lowering the signal-to-background ratio. – The reporter fluorophores (dyes) must have different emission spectra. Fluorophores from a list of those compatible with the instrument at hand should be chosen for different channels. Probes labeled with FAM and HEX are cleaved more efficiently than probes labeled with ROX or CY5. – It is best to label with a brighter dye the probe having lower Tm or GC content or containing an A/T allele, which binds worse to the template (for example, FAM gives a stronger signal than HEX does).

Use of locked nucleic acid (LNA) modifications in oligonucleotides

Commercially available TaqMan probes can be conjugated to a minor groove binder (MGB) motif, e. g., dihydrocyclopyrroloindole tripeptide (DPI3), to increase the probe’s binding affinity for the target sequence. This approach allows to increase melting temperature of the probe without increasing its length, thus improving discrimination between the complementary probe and noncomplementary probe.

Commercially available alternatives to this technology are locked nucleic acids (LNAs or bridged nucleic acids, BNAs), which are analogs of RNA with ribose locked in the 3′-endo conformation due to a 2′-O, 4′-C methylene bridge. The presence of these modified bases in the oligonucleotide enhances the thermal stability and specificity of hybridization (Owczarzy et al., 2011). Such nucleotides are usually marked as [+X] or +X (where X = A, T, G, or C). By replacing individual nucleotides in a probe with their LNA analogs, it is possible to make the probe itself shorter, and the contribution that the polymorphic site makes to the total Tm is greater, which will facilitate the discrimination of alleles. Typically, a modification of a single nucleotide at the SNP position is made, but for each sequence, effects of different variants may differ (You et al., 2006).

The choice of tools for calculating Tm of oligonucleotides with an LNA modification is narrower than in the case of unmodified bases; you can use OligoEvaluator services (http:// www.oligoevaluator.com/LoginServlet) or the OligoAnalyzer Tool (https://www.idtdna.com/calc/analyzer).

Unfortunately, there are currently no available services for calculating the Tm difference between the fully paired duplex and the heteroduplex containing unpaired bases for oligonucleotides with an LNA modification. Previously, the OligoAnalyzer Tool has allowed for such calculations, but due to low accuracy, this option has been removed. To roughly estimate the effect of LNA on mismatches, you can use data from articles on the thermodynamics of oligonucleotides with LNA modifications. In some cases, an LNA modification even reduces the match vs. mismatch difference as compared to the unmodified oligonucleotide; therefore, these modifications require caution (You et al., 2006).

Design example

By changing the amplicon length, melting temperature, GC content, and positions and lengths of primers and probes, it is possible to obtain combinations that satisfy the above criteria and to select the best one. SNP-containing sequences being analyzed do not always permit designing primers and probes that meet all the aforementioned criteria, but this does not mean that the selected assay will not work in practice

Let’s consider the algorithm for designing primers and probes for analysis of the rs11121704 polymorphism

**Fig. 3.**
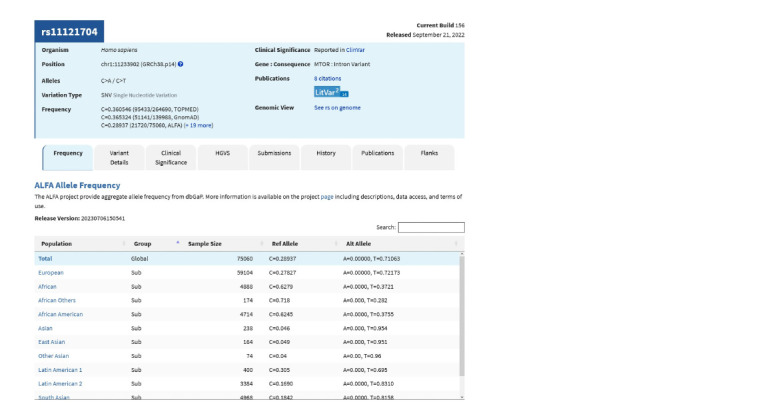
Basic information about the rs11121704 variant: chromosome, position, genome assembly, and frequency of nucleotide substitutions

1. Find the polymorphism in the dbSNP database (https:// www.ncbi.nlm.nih.gov/snp/), and go to the page with a detailed description (https://www.ncbi.nlm.nih.gov/snp/ rs11121704).

2. Find the substitution you need, and pay attention to the genome assembly for which the position is specified. For example, in our case, the latest one is currently GRCh38. At the next stage, we need information about the chromosome (NC_000001.11), the position on it (11233902), and the substitution (C>T). One rsID can correspond to several variants at one locus; all of them are listed on the page (C>A/C>T). Typically, most people have either the reference allele or the most common alternative allele. The necessary information can be found on the “Frequency” tab (Fig. 3).

In this genotyping method, we regard all polymorphisms as biallelic, and we are usually interested in the most common substitution at a given position because variants with a near-zero frequency can occur only in large study populations

3. To select primers, we use the open online resource Primer- Blast (https://www.ncbi.nlm.nih.gov/tools/primerblast/)

In the “Enter accession” field, specify the chromosome with the polymorphism you are interested in (NC_000001.11).

In the “Range” fields, we indicate the boundaries within which the forward and reverse primers should lie near the SNP position (11233902). We set the boundaries of the primerbinding site no closer than 15 nucleotides to the SNP (because otherwise, primer landing may be impeded by the probe) and not farther than 200 (so that the amplicon is not too long and the reaction efficiency is higher): the forward primer from 11233702 (11233902-200) to 11233887 (11233902-15), and the reverse one from 11233917 (11233902+15) to 11234102 (11233902+200). “PCR product size” is set to 100–250.

In the “Database Refseq” field, choose “Refseq representative genomes” or “Genomes for selected organisms (primary reference assembly only)”. Both databases contain primary assemblies of chromosomal sequences with minimal redundancy, and “representative genomes” also include alternative loci and mitochondrial genomes, if available.

The “Advanced parameters” option provides access to additional parameters, among which, we are interested in “Primer GC content (%)”, for which we set the range to 40–60 %.

**Fig. 4.**
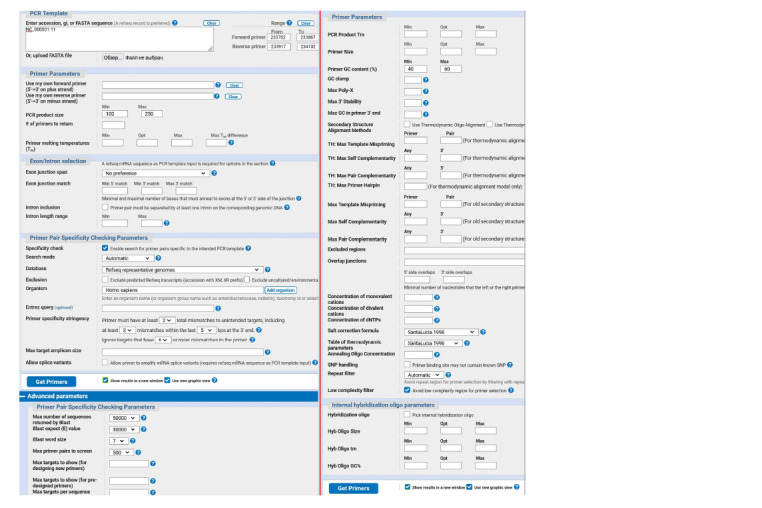
An example of settings for designing primers in the Primer-Blast tool.

We leave the remaining parameters unchanged. Before clicking the “Get primers” button, select the “Show results in a new window” option so that after the results are displayed, it is easier to change individual launch parameters for a second search (Fig. 4).

4. Go to the page with the primer search results. We select primers that are suitable for the position, Tm, and specificity. You can work with nonspecific primers, but you must then check that the probe binds only to the specific amplicon and not to side products.

**Fig. 5.**
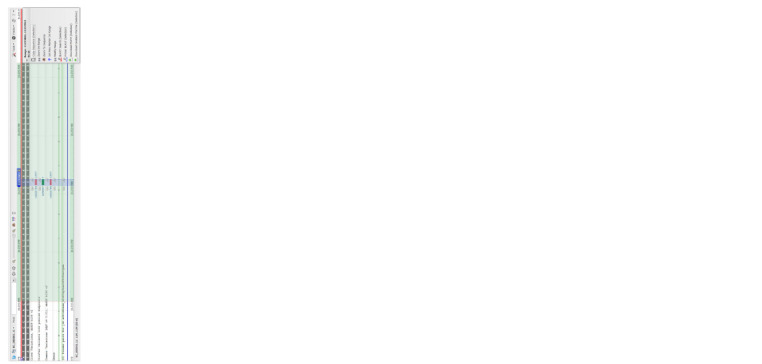
The selected primers in the Primer-Blast tool give a specific target PCR product 130 nucleotides long containing SNP rs11121704, and one potential PCR product 1,186 nucleotides long, which should not form under normal conditions

We chose the sequence 5′-TTTTTCCTCATTTTGGGC GA-3′ for the forward primer and 5′-TATCAGTTGCAG GAAAGTGC-3′ for the reverse primer. The “results” page shows that the selected primers give a target specific product 130 nucleotides long (Fig. 5). There is also one potential nonspecific PCR product with a length of 1,186 nucleotides, which will not be synthesized due to incomplete complementarity of the binding sites to the primer sequences (Fig. 5).

**Fig. 6.**
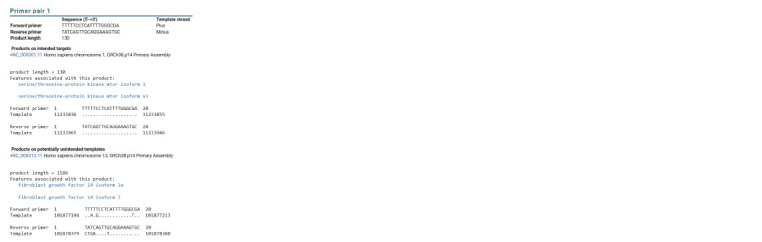
A nucleotide sequence that flanks the SNP and does not overlap with other known polymorphic sites

5. Next, in the “Tracks” option, select the “Configure Tracks” suboption, find and check the boxes “Common variations (MAF>=0.01)”, “Cited Variations”, and “ClinVar variants with precise endpoints” and add them to the display with the “Configure” button. A probe should not overlap with polymorphisms other than the one of interest.

6. In the search results, select the sequence area around the SNP (nucleotides –20...+20) and click “copy sequence (selection)” (Fig. 6).

Additionally, build a complementary strand to this sequence. This can be done manually or use any available service, for example, (https://www.bioinformatics.org/sms/ rev_comp.html).

Forward strand sequence: 5′-TTCTCCTTTCCAAACATCTG(C)GATGATGTGCC TGAAGCATT-3′ Reverse strand sequence: 5′-AATGCTTCAGGCACATCATC(G)CAGATGTTTGG AAAGGAGAA-3′ The position of the SNP in question is indicated in brackets.

7. Within the sequence near the SNP, select a fragment of suitable length and composition. Based on the GC content, it is worthwhile taking the reverse strand sequence, because in this case, there will be more Cs than Gs in the probe: 5′-CAGGCACATCATC(G)CAGATGTTT-3′ Given that we are not taking the strand in which the SNP is shown to be located, you should remember that for our sequence, the C>T substitution in the reverse strand corresponds to the G>A substitution. We select the boundaries of the second probe so as to equalize the probes’ Tm: 5′-CAGGCACATCATC(A)CAGATGTTTG-3′

8. It is recommended to check Tm by means of several services and to average it (see the Table). For example, we used Oligo Calc (http://biotools.nubic. northwestern.edu/OligoCalc.html), OligoEvaluator (http:// www.oligoevaluator.com/LoginServlet), and OligoAnalyzer Tool (https://www.idtdna.com/calc/analyzer).

**Table 1.**
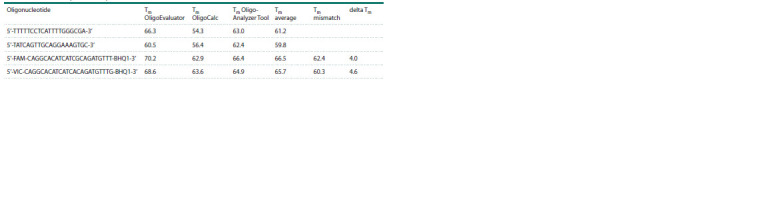
Main characteristics of primers and probes for rs11121704 Notе. Melting temperatures (Tm) predicted by several services and their averages are listed. The Tm mismatch is the melting temperature of the probe in the noncomplementary duplex with the template. “delta Tm” is the difference between “Tm (Oligo-Analyzer Tool)” and “Tm mismatch” .

9. To compare the melting temperatures between the fully complementarily bound probe and the probe forming an unpaired base, select the “Tm mismatch” option in the OligoAnalyzer Tool.

For the first probe CAGGCACATCATC(G)CAGATGTTT, the mismatch is the nucleotide complementary to the second probe (CAGGCACATCATC(A)CAGATGTTTG), i. e., select the letter “T”, and click “Use Exact Complement Tm” and “Calculate”. The greater the difference in the Tm between thefully complementary oligonucleotide and the probe having the noncomplementary base (“deltaTm”) (and accordingly, the lower the proportion of the bound mismatched probe compared to the fully complementary probe at the probe annealing stage), the more accurate the allele discrimination will be. 

10. To check the specificity of the newly designed probes, we use the Blast service (https://blast.ncbi.nlm.nih.gov/ Blast. cgi?PROGRAM=blastn&PAGE_TYPE=BlastSearch& LINK_LOC=blasthome).

In the “Enter accession number(s), gi(s), or FASTA sequence( s)” field, insert the probe sequence; “Database” should be “Refseq representative genomes”, “Organism” should be “human (taxid:9606)”. Select option “Show results in a new window,” and press the “Blast” button. It is important for us that the probe does not bind to nonspecific PCR products (if any exist) and binds to the single region of the target amplicon.

11. It is worthwhile to check primers and probes for complementarity to each other and for self-complementarity and hairpin formation in the OligoAnalyzer Tool software according to their recommendations (https://www.idtdna.com/pages/ education/decoded/article/designing-pcr-primers-and-probes). In this software, we check a parameter called ΔG (change in Gibbs free energy) of secondary-structure formation. At ΔG values more positive than –9 kcal/mol, secondary structures do not have a significant effect on PCR, and values greater than zero indicate that under these conditions, secondary structures do not form (https://www.gene-quantification.de/ oligo_architect_glossary.pdf). Therefore, when checking the primers and probes, we select those with ΔG ≥ –9 kcal/mol for potential secondary structures

## PCR execution and choosing PCR conditions

Fluorophore-labeled probes should be stored in the dark to avoid photobleaching (https://assets.thermofisher.com/TFSAssets/ LSG/Application-Notes/cms_043004.pdf).

Fluorophore-labeled probes should be stored in the dark to avoid photobleaching (https://assets.thermofisher.com/TFSAssets/ LSG/Application-Notes/cms_043004.pdf).

Mix all the listed components, except for the DNA sample, in a microtube while taking into consideration the number of the samples (with a 10 % excess of the mix). Place the DNA samples directly into wells of the PCR plate, then add 18 μl of the mix into each well, vortex, and centrifuge down.

Check the performance of the new primers and probes by means of several DNA samples and select optimal annealing temperatures first. Optimal concentrations of primers and probes may also differ from those given above, but the final concentrations of probes are usually at least 2 times lower than those of primers (https://www.bioline.com/mwdownloads/ download/link/id/3301//p/i/pi-50201_sensifast_probe_hi-rox_ one-step_kit_v11.pdf).

**Fig. 7.**
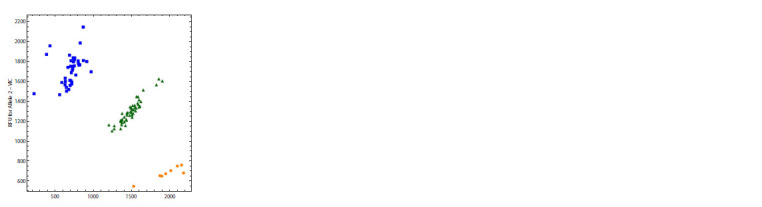
The result of allelic discrimination using probes for SNP rs11121704. Orange dots represent homozygotes of the reference allele (C/C), green triangles represent heterozygotes (C/T), and blue squares represent homozygotes of the alternative allele (T/T).

PCR program:

1. Initial denaturation, 95 °C for 3 min, 2. Amplification and detection (40 cycles): denaturation, 95 °C for 10 s, primer annealing and elongation with signal detection, 60 °C for 30 s. The outcome of PCR with the chosen primers and probes for SNP rs11121704 is presented in Figure 7.

Because Tm of an oligonucleotide is the temperature at which half the population of oligo molecules is molten and half is double-stranded, the recommended annealing temperature should be approximately 5 °C lower than the lower Tm between the two primers, because under such conditions, both primers will bind almost completely to the complementary strands. In practice, due to possible inaccuracy of Tm calculation or discrepancies between the reaction conditions and the conditions for which the calculation was performed, the optimal temperature is selected empirically. We recommend checking the interval [Tmav – 5 °C…Tmav + 5 °C], where Tmav is the average Tm value of the two primers. We also recommend choosing conditions for two PCR mix versions: with probes or with an intercalating dye, for example, SYBR Green. The melting curve plot will identify possible nonspecific PCR products.

Elongation typically takes ~1 min per 1,000 bp. Often, if the annealing temperature is greater than 60 °C, this step is combined with the previous one, and elongation occurs at the annealing temperature. Although the temperature optimum for most Taq polymerases is approximately 75–80 °C, elongation cannot occur at temperatures higher than the melting point of the probes

## Conclusion

Genotyping by allele-specific PCR is an effective and accurate way to detect genetic variants. Advantages of this method are its specificity, sensitivity, low cost, and quick results. It makes it possible to distinguish different alleles in the genome by one-step PCR without additional product separation steps; accordingly, it is particularly useful for genetic association studies in molecular genetics and medicine.

Thanks to developments in technologies for the synthesis of oligonucleotides and improvements in methods for designing primers and probes, we can expect expansion of the possibilities offered by this approach in the diagnosis of hereditary diseases. In this article, we discussed in detail the criteria and conditions for optimizing successful design primers and oligonucleotide probes for allele-specific PCR. We hope that the presented protocol will enable research groups to independently design their own effective assays for testing for polymorphisms of interest.

## Conflict of interest

The authors declare no conflict of interest.

## References

Broccanello C., Chiodi C., Funk A., McGrath J.M., Panella L., Stevanato
P. Comparison of three PCR-based assays for SNP genotyping
in plants. Plant Methods. 2018;14:28. DOI 10.1186/s13007-018-
0295-6

Debode F., Marien A., Janssen E., Bragard C., Berben G. The influence
of amplicon length on real-time PCR results. Biotechnol. Agron.
Soc. Environ. 2017;27(1):3-11. DOI 10.25518/1780-4507.13461

Huang Q., Li Q. Characterization of the 5ʹ to 3ʹ nuclease activity
of
Thermus aquaticus DNA polymerase on fluorogenic double-stranded
probes. Mol. Cell. Probes. 2009;23(3-4):188-194. DOI 10.1016/
j.mcp.2009.04.002

Hui L., DelMonte T., Ranade K. Genotyping using the TaqMan assay.
Curr. Protoc. Hum. Genet. 2008;56(2):2.10.1-2.10.8. DOI 10.1002/
0471142905.hg0210s56

Kalendar R., Shustov A.V., Akhmetollayev I., Kairov U. Designing
allele-specific competitive-extension PCR-based assays for highthroughput
genotyping and gene characterization. Front. Mol. Biosci.
2022;9:773956. DOI 10.3389/fmolb.2022.773956

Owczarzy R., You Y., Groth C.L., Tataurov A.V. Stability and mismatch
discrimination of locked nucleic acid-DNA duplexes. Biochemistry.
2011;50(43):9352-9367. DOI 10.1021/bi200904e

Ranade K., Chang M.S., Ting C.T., Pei D., Hsiao C.F., Olivier M.,
Pesich R., Hebert J., Chen Y.D., Dzau V.J., Curb D., Olshen R.,
Risch N., Cox D.R., Botstein D. High-throughput genotyping with
single nucleotide polymorphisms. Genome Res. 2001;11(7):1262-
1268. DOI 10.1101/gr.157801

You Y., Moreira B.G., Behlke M.A., Owczarzy R. Design of LNA
probes that improve mismatch discrimination. Nucleic Acids Res.
2006;34(8):e60. DOI 10.1093/nar/gkl175

